# The Potential Use of Orange and Banana Peels to Minimize the Toxicological Effects of Silver Nanoparticles in *Oreochromis Niloticus*

**DOI:** 10.1007/s00128-022-03488-5

**Published:** 2022-03-11

**Authors:** Amr Adel Abdel-Khalek, Aliaa Hamed, Wafaa S.F. Hasheesh

**Affiliations:** 1 Department of Zoology, Faculty of Science, Cairo University, Giza, Egypt; 2 Department of Biology, Basic Science Center, Misr University For Science and Technology (MUST), Giza, Egypt

**Keywords:** Silver nanoparticles, Hematological biomarkers, Histopathology, Bioremediation, Metals accumulation, Orange and banana peels, *Oreochromis niloticus*

## Abstract

To evaluate the effectiveness of orange peels (OP) and banana peels (BP) in reducing the toxicity of silver nanoparticles (Ag-NPs), *Oreochromis niloticus* were exposed to Ag-NPs, Ag-NPs + OP, and Ag-NPs + BP for 24, 48, and 96 h. Time-dependent toxicological impacts of Ag-NPs were recorded. The maximum Ag accumulation was in hepatic and renal tissues after 96 h. A marked decrease in red blood cell count, hemoglobin content, hematocrit ratio, and mean corpuscular hemoglobin concentration was observed after 48 and 96 h of Ag-NPs exposure. Silver accumulation resulted in severe histological alterations (ex: congestion, vacuolization, and necrotic degeneration) in gills, livers, and kidneys. The adsorptive capacity of both peels could reduce the bioavailability of Ag-NPs as indicated by decreased Ag content in tissues, insignificant change in the hematological parameters with control groups, and regressive histological alterations based on the frequency of alterations’ existence and the extent of affected parts.

## Introduction

The toxicological impacts of metallic nanoparticles (NPs) in aquatic bodies are still questionable compared to other aquatic pollutants (Khan et al. 2018). The NPs could reach aquatic bodies during their applications through the waste discharge in many industries (Mao et al. 2018). A strong emphasis was given to aquatic environments because they were and still are the main gathering point for NPs’ dispersion into other environmental resources (Tunçsoy et al. 2017). For this reason, different studies were designed to find suitable strategies to minimize the hazardous effects of those NPs on aquatic ecosystems. The production and application of silver nanoparticles (Ag-NPs) have been increased with an expected elevation rate of up to 63% of the global nanomaterial products by 2024 (Inshakova and Inshakov 2017). The excessive utilization of Ag-NPs is mainly due to their unique antimicrobial, optical, electrical, and magnetic properties. Therefore, silver NPs are commonly used in cosmetics, textiles, toothpaste, shampoos, paints, washing machines, and food supplements (Mao et al. 2018). The uncontrolled consumption of Ag-containing products acts as a double-edged sword as their widespread use elevates the discharge of Ag-NPs into the aquatic habitat. In the near future, aquatic organisms will suffer if the Ag-NPs toxicity is not mitigated (Vali et al. 2020). Several harmful effects such as cytotoxicity, oxidative stress, histological alterations, and inflammatory responses were recorded in fish after their exposure to Ag-NPs (Mahmoud et al. 2019). The toxic effects of Ag-NPs were related to various characteristics such as particle size, surface area, surface chemistry, coagulation, and aggregation state. Moreover, the existence of Ag-NPs in a colloidal form increased the accumulation capacity of these particles into aquatic organisms (Inshakova and Inshakov 2017). The absorbed Ag-NPs can migrate through the bloodstream to several tissues, causing a variety of hematological and structural damages. Consequently, hematological endpoints in the aquatic environment are being utilized to track the toxic effects of NPs on different blood components (Thummabancha et al. 2016). Various hematological parameters and blood indices have become significant health indicators for many fish and offer a major role in understanding their normal or pathological status (Abdel-Khalek et al. 2016). Furthermore, the accumulation of NPs could potentially cause structural damage in certain tissues. The histological biomarker was considered a sensitive tool in toxicological fields as it showed the direct influence of NPs on the regular architecture of cells and tissues (Khan et al. 2018). Therefore, using hematological and histological endpoints can give a real picture of the health status of the studied fish. Nile tilapia; *Oreochromis niloticus*, is capable of adapting to and resisting various unsuitable conditions. Therefore, this fish species is considered a good bioindicator representing the impact of many toxicants on animal health (Abdel-Khalek et al. 2018). Several techniques are available to purify contaminated water, but treatment facilities are difficult and expensive. Thus, different technologies were developed depending on ecofriendly materials like agricultural wastes due to their high availability, simple synthesis, and low cost. Many agricultural wastes are mainly rich in lignin and cellulose, so they can be used as alternative adsorbents due to their specific structure and chemical properties (Singh et al. 2018). Moreover, specific functional groups such as alcohol, phenol, aldehyde, carboxyl, and ketone groups are present in their polymer chains which give them a high adsorption capacity for metals and organic compounds (Bhatnagar et al. 2015). In this context, fruit peels can be used as potential sources of adsorbent materials for wastewater treatment. Orange peels (OP) and banana peels (BP) residues can be processed and converted to be biosorbents (biological materials used to eliminate contaminants passively from the surrounding environment) due to their chemical composition (cellulose and other polysaccharides richness), large surface areas, high swelling capacities, and excellent mechanical strengths that give them a great potential to adsorb harmful contaminants (Annadurai et al. 2003; Kelly-Vargas et al. 2012). In this regard, the present approach has two simultaneous aims (1) evaluating the accumulation potency of Ag-NPs and their effects on different hematological and histological biomarkers in *O. niloticus* (2) using OP and BP as bio-sorbents to reduce Ag-NPs’ bioavailability in water and, consequently, their toxicity.

## Materials and methods

### Ag-NPs preparation and characterization

The silver NPs with a product number of 576,832 were purchased from Sigma-Aldrich, St. Louis, MO, USA. As provided by the manufacturer, the molecular weight of those NPs is 107.87 g/mole, particle sizes are less than 100 nm with 99.5% purity. As detailed in our previous study Abdel-Khalek et al. (2021), structural studies were done to assess the actual particle size, surface charge, and aggregation behavior in water. The half-lethal concentration of Ag-NPs was previously determined by Sarkar et al. (2015) using *Oreochromis niloticus* as an animal model to be 8 mg/L. In the present study, ^1^/_2_ LC_50_/96 h. (4 mg/L) was used as an actual concentration by dispersing the dry nano-powders in dechlorinated water (pH: 7.4) then ultrasonicated for 1 h (100 W, 40 kHz) using an ultrasonic homogenizer (BioLogics, Inc., Manassas, VA, USA) to increase NPs dispersion in water. The nominal concentration of Ag in water was determined by inductively coupled plasma (ICP-AES), Thermo Sci, model: iCAP6000 series to be 87 ± 2% of the actual concentration.

### Preparation of OP and BP

Both peels were washed with deionized water to remove any debris, then put in an oven at 80 °C for one day to confirm the complete dryness. The peels were used without any physical or chemical modification. After that, the adsorbents (OP and BP) were ground by a grinder and sieved to get particle sizes between 1 and 5 mm as recommended by Annadurai et al. (2003). In fish aquaria, absorbents were separated by a porous mesh (less than 1 mm) to enable the passage of Ag-NPs into the absorbents and, at the same time, prevent absorbents from escaping into the water to avoid their ingestion by fish. A higher dosage of adsorbent allows more active sites for potential interaction with the target contaminants in the medium. So, the absorbent concentration was 40 mg/L (10 times the used NPs concentration) as recommended by Akpomie and Conradie (2020).

### Experimental design

Adult male *Oreochromis niloticus* (bodyweight ranged from 41.91 to 50.17 g and body length ranged from 10 to 15 cm) were obtained from an unpolluted fish farm located at Kafr El-Sheikh governorate, Egypt. Fish were transported to the ecology laboratory of the Faculty of Science, Cairo University, with good aeration conditions in large plastic containers. In glass aquaria (40 × 70 × 26 cm), fish were divided (7 fish/aquarium) to adapt for 14 days in 50 L of aerated and dechlorinated tap water. Water parameters were checked daily and kept as 25 ± 1 °C for temperature, 7.1–7.8 mg/L for dissolved oxygen, and 7.2–7.4 for pH. During the adaptation period, fish were fed twice daily with commercial food pellets (20% crude protein, 4% crude fat, 5% crude fiber, 12% crude ash, and10% crude moisture). Fish were grouped into: control groups (dechlorinated tap water), Ag-NPs (4 mg/L) exposed groups, Ag-NPs (4 mg/L) + OP (40 mg/L) groups, and Ag-NPs (4 mg/L) + BP (40 mg/L) groups for 24, 48, and 96 h.

### Fish sampling

At the end of each experimental period, blood samples from the caudal vein of seven fish/group were withdrawn by heparinized syringes and clove oil (25 mg/L) as an anesthetic agent. The fish were dissected, then the targeted tissues were isolated for further examination.

### Silver concentrations in different fish tissues

Silver bioaccumulation levels in the liver, gills, kidneys, skin, and muscle tissues of the studied fish were measured after 24 h., 48 h., and 96 h. using inductively coupled plasma (ICP-AES), Thermo Sci, model: iCAP6000 series. The limit of detection of Ag metal was 0.3 µg/L. All measuring procedures were done according to APHA (2005). Based on the dry ashing method (Neugebauer et al. 2000), the isolated tissues were dried at 80 °C for 8 h. The samples were acid digested by concentrated nitric acid and perchloric acid (4:1, v-v). The digested tissues were diluted by deionized water to a known volume in volumetric flasks. The procedural blanks (all used reagents without sample) were aspirated before sample analysis to correct the background absorption. A standard reference material (Lake Superior fish 1946 NIST, National Institute of Standards and Technology, USA) was used to validate the analysis procedure. The recorded metal recovery range was 94–106%. During quantifying silver concentrations, a standard solution with a known Ag concentration was measured (every 5 samples) to check the accuracy of the measurement process.

### Hematological parameters and indices

The red blood cells (RBCs) were counted after blood dilution with 0.7% NaCl using the improved Neubauer hemocytometer as described by Dacie and Lewis (1991). The concentration of hemoglobin (Hb) was measured according to Drabkin (1964), who was based on the conversion of Hb into colored cyanomethemoglobin. Then, cyanomethemoglobin (equivalent to Hb content) was measured spectrophotometrically at 540 nm. The values of hematocrit (Hct) were calculated using heparinized capillary tubes and Hct centrifuge at 3000 r.p.m. for 15 min. The percentage volume of the RBCs to total blood volume was estimated by a hematocrit reader.

As described by Gupta (1977), all blood indices were calculated as shown:

The mean corpuscular volume (MCV)=$\frac{\text{H}\text{c}\text{t} \times 10}{\text{R}\text{B}\text{C}\text{s} (\text{m}\text{i}\text{l}\text{l}\text{i}\text{o}\text{n}/\text{m}\text{m}3) }$

The mean corpuscular hemoglobin (MCH)=$\frac{\text{H}\text{b}(\text{g}\text{m}/\text{d}\text{l}) \times 10}{\text{R}\text{B}\text{C}\text{s} (\text{m}\text{i}\text{l}\text{l}\text{i}\text{o}\text{n}/\text{m}\text{m}3) }$

The mean corpuscular hemoglobin concentration (MCHC) =$\frac{\text{H}\text{b} (\text{g}\text{m}/\text{d}\text{l}) \times 100}{\text{H}\text{c}\text{t} }$

### Histological study

The tissues named gills, liver, and posterior kidney were isolated and washed with 0.7% NaCl solution then preserved in Bouin’s fixative. Based on the method described by Bernet et al. (1999), all tissues were processed, sectioned at 4 μm, and then stained by hematoxylin and eosin. For each group, fourteen specimens from seven different fish were examined for each tissue (2 slides/fish), and the histopathological alterations were recorded.

### The SEM/EDS spectral and map analysis of OP and BP surfaces

After each study period, both OP and BP were collected from the aquaria and scanned to check the adsorption efficiency of Ag-NPs on their surfaces using scanning electron microscopic (SEM) models FEI Inspect S 50-Netherlands and Energy Dispersive X-Ray Spectroscopy (EDS) attachment with Bruker AXS-Flash Detector, 410-M-Germany.

### Statistical analysis

The data were represented as mean ± standard error. All data were statistically analyzed using analyses of variance (ANOVA) and Duncan’s multiple ranges to estimate the significant difference (p < 0.05) between the same experimental group at different times and between the different experimental groups at the same experimental period. The statistical analysis was done using Statistical Processor Systems Support, SPSS software, version 16.0, IBM, Chicago, IL, USA.

## Results

### Characterization of Ag-NPs

**Fig. 1:**
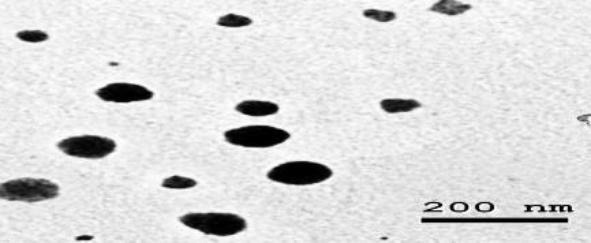
Characterization of Ag-NPs as shown in our previous study Abdel-Khalek et al. (2021)

The characterization data of Ag-NPs revealed that the examined nano-sized particles (41.3 ± 5.2 nm) had a spherical shape, low aggregation affinity, and a good dispersion pattern in water as confirmed by zeta potential (− 13.8 mV) and DLS (84.23 nm) results (Fig. 1).

### Silver concentrations in different fish tissues

**Fig. 2:**
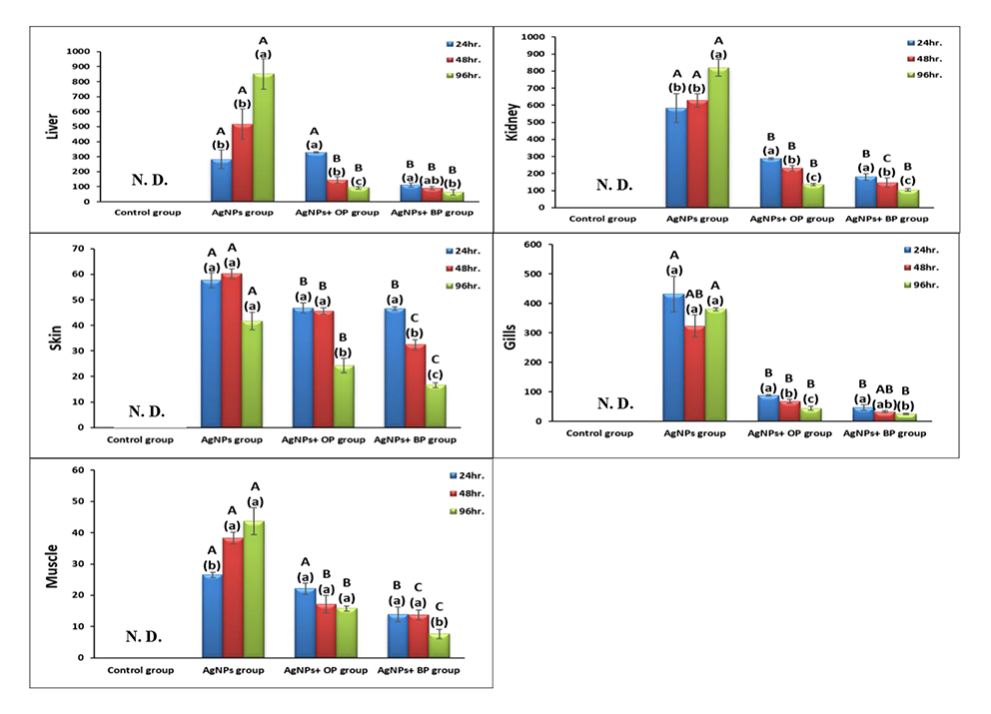
Silver accumulations (mg/Kg dry wt.) in vital tissues of studied fish groups after 24 h., 48 h., and 96 h. (Data are represented as means of seven samples in each time interval of each group ± SE; The small letters represent Duncan’s test (p < 0.05) between different experimental periods within the same group. Columns with the same small letters are not significantly different; otherwise, they do; The capital letters represent Duncan’s test (p < 0.05) between the same experimental periods among different groups. Columns with the same capital letters are not significantly different; otherwise, they do; The letters are arranged in descending order as A, B, and C; N. D.: Not detected)

Concerning the accumulation capacity of Ag-NPs in the studied tissues (Fig. 2), there were significant differences (P < 0.05) among tissues and studied groups. In comparison with the control groups, the Ag concentrations showed significant increases in the tissues of all studied groups, with maximum elevation in Ag-NPs exposed groups at all studied periods. The accumulation potency of different tissues toward Ag-NPs was maximized in the liver and kidney, followed by the gills, while muscular tissues showed the minimum Ag content in all studied groups. There was a significant decrease in Ag accumulation levels in OP and BP treated groups at all studied time intervals.

### Hematological parameters and indices

**Fig. 3:**
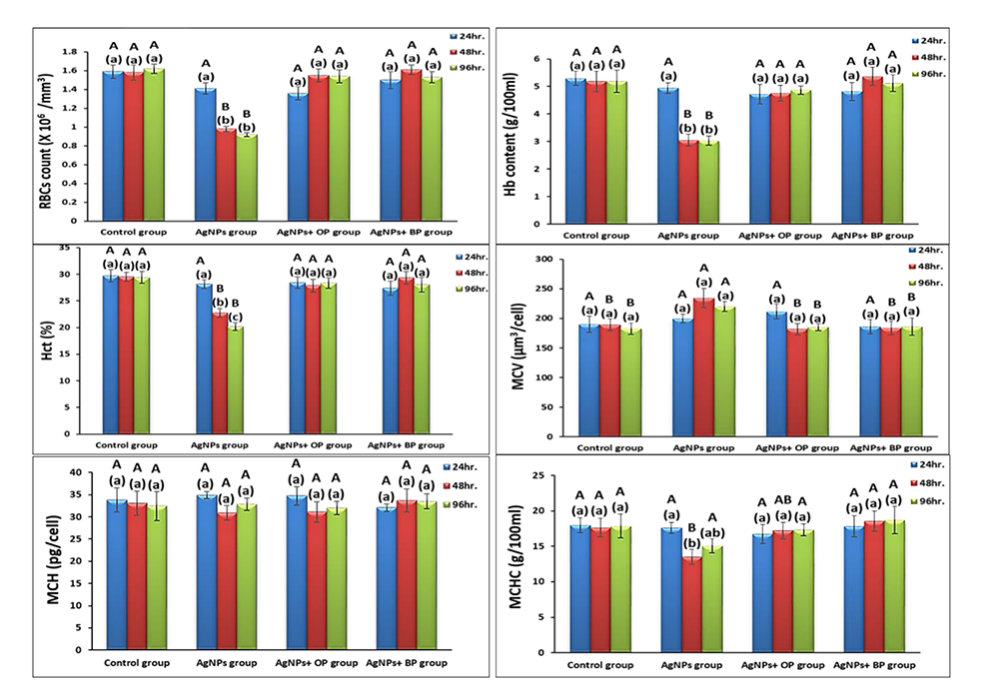
The effect of exposure to Ag-NPs, Ag-NPs with OP, and Ag-NPs with BPs on some hematological parameters and indices. (Data are represented as means of seven samples in each time interval of each group ± SE; The small letters represent Duncan’s test (p < 0.05) between different experimental periods within the same group. Columns with the same small letters are not significantly different; otherwise, they do; The capital letters represent Duncan’s test (p < 0.05) between the same experimental periods among different groups. Columns with the same capital letters are not significantly different; otherwise, they do; The letters are arranged in descending order as A, B, and C; N. D.: Not detected)

As shown in Fig. 3, the studied hematological parameters (RBCs, Hb, and Hct) showed a remarkable decrease (Duncan’s test capital letters) after 48 and 96 h. of exposure to Ag-NPs. While the hematological results of fish that were exposed to Ag-NPs with OP, and Ag-NPs with BPs showed insignificant changes compared to the control fish at all studied periods. Regarding the calculated blood indices, there were insignificant changes among the different exposed fish groups all over the studied periods compared with the control groups. The values of MCV and MCHC showed a significant change in Ag-NPs exposed fish after 48 and 96 h. of exposure that indicated an enlargement in RBCs to some extent.

### Histological analysis

#### Gills

**Fig. 4:**
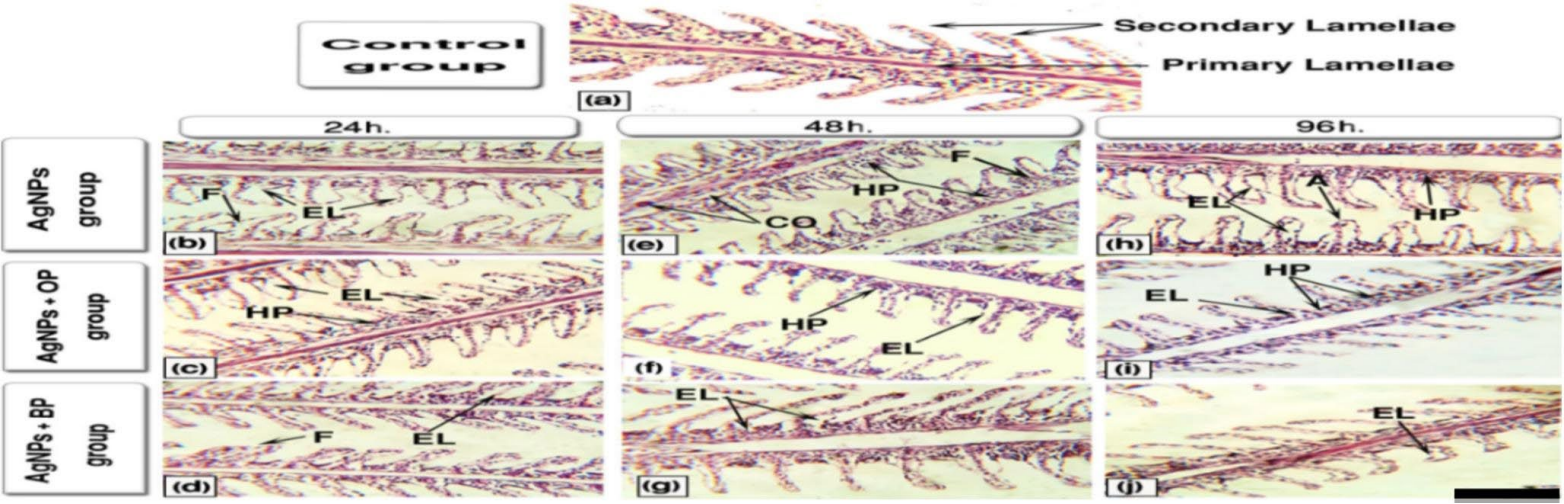
Representative histological alterations in gills of the studied fish (Scale bar = 100 μm)

The histological structure of the control groups (Fig. 4a) showed well-structured gill filaments, primary lamella, well-separated secondary lamellae with flat epithelial cells, and chloride cells at their bases.

While sections obtained from fish that were exposed to Ag-NPs after 24 h., 48 h., and 96 h. of exposure (Fig. 4b and e, and 4 h) showed histological changes as epithelial lifting (EL) along secondary lamellae; fusion in secondary lamellae (F); congestion in the lamellar blood vessels (CO); hyperplasia (HP) in the epithelia of the primary lamellae; aneurysm (A) [outward bulging caused by an abnormal weak spot on a blood vessel wall] at the tips of secondary lamellae. Sections of fish exposed to Ag-NPs with OP (Fig. 4c and f, and 4i) and with BP (Fig. 4d g, and 4j) showed less noticeable alterations along with all studied periods. The epithelial lifting became limited to the bases or tips of secondary lamellae. Also, mild hyperplasia and fusion of lamellae were recorded.

#### Liver

**Fig. 5:**
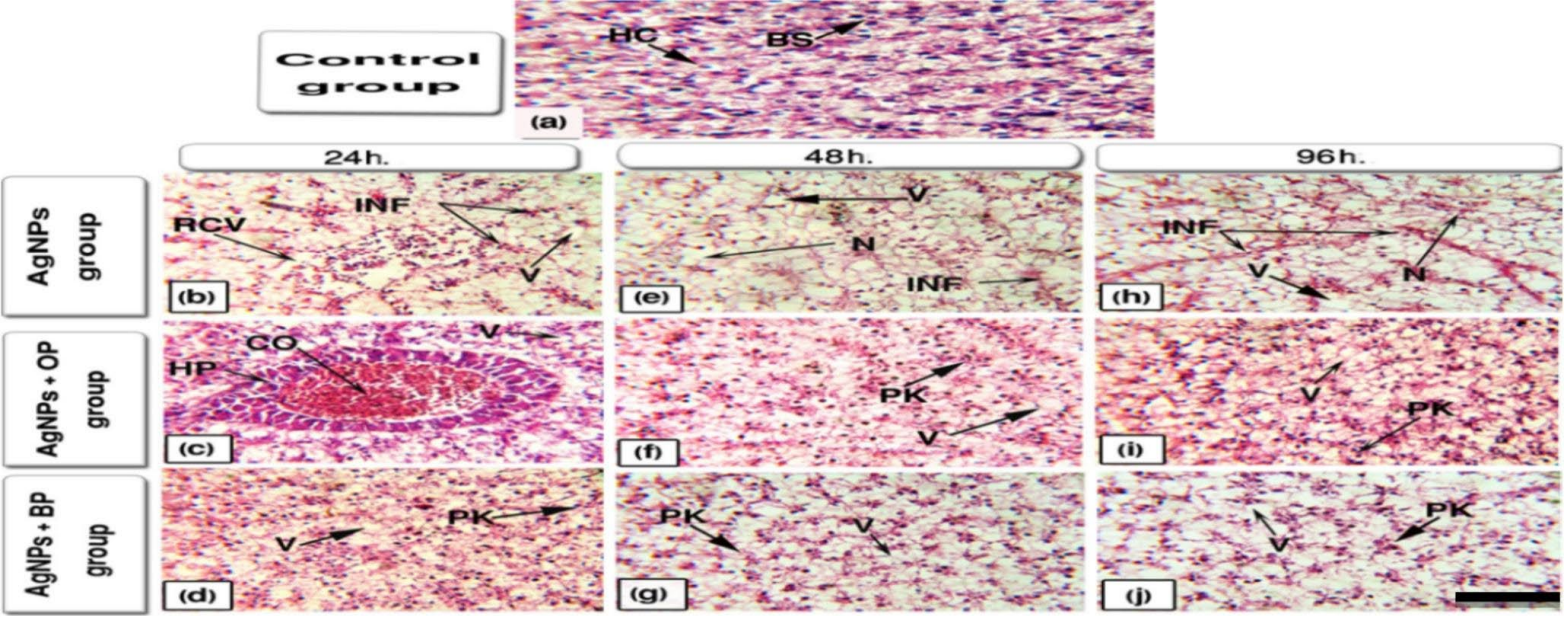
Representative histopathological alterations in liver tissues of the studied fish (Scale bar = 100 μm)

The hepatic tissues of the control fish (Fig. 5a) showed the normal histological structure of hepatocytes (HC) with normal central or sub-central sphere-shaped nuclei and homogenous cytoplasm.

While, liver sections of fish that were exposed to Ag-NPs without any water treatment after 24 h., 48 h., and 96 h. of exposure (Fig. 5b and e, and 5 h) showed marked deteriorations in liver architecture as rupture of central vein (RCV); infiltration of blood cells (INF); vacuolization (V); necrosis (N). Whereas the hepatic tissues of fish that were exposed to the same concentration of Ag-NPs with OP in water (Fig. 5c and f, and 5i) and BP in water (Fig. 5d g, and 5j) showed some blood congestion (CO) in hepatopancreatic tissue (HP); vacuolization (V); pyknotic nuclei (PK) [chromatin condensation in the nuclei suffered from necrosis or apoptosis].

#### Kidney

**Fig. 6:**
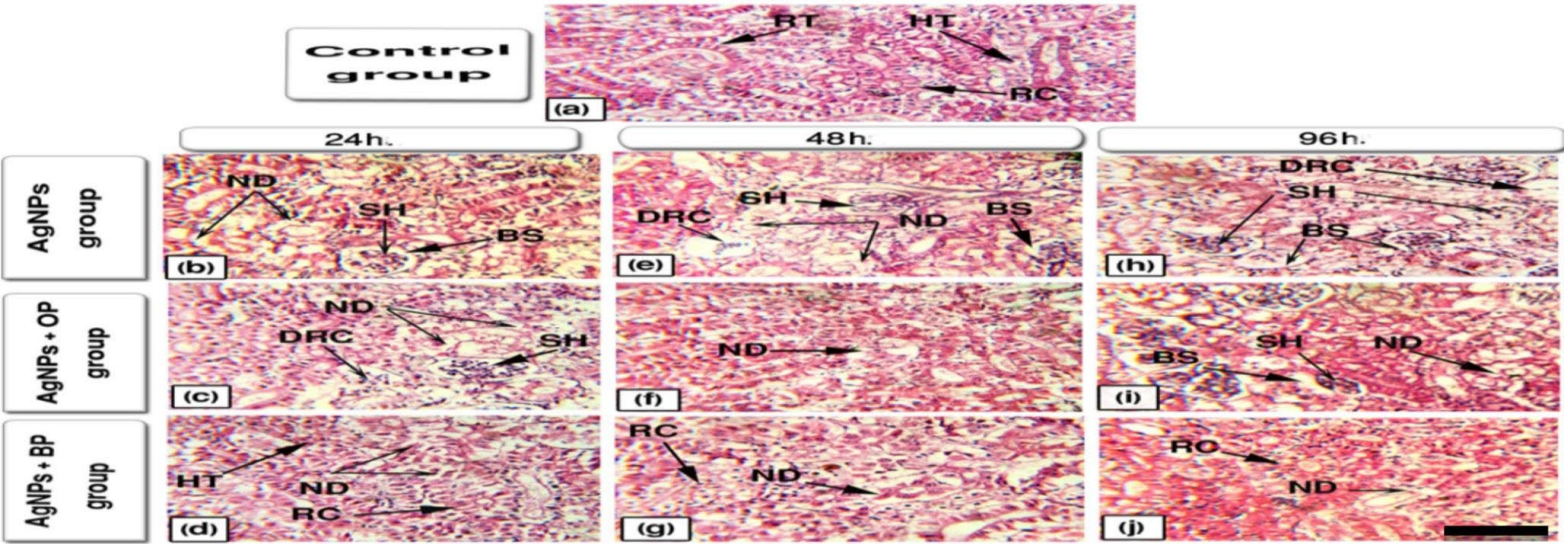
Representative histopathological alterations in renal tissues of the studied fish (Scale bar = 100 μm)

Kidneys of the control groups (Fig. 6a) showed regularly formed renal corpuscles (RC) and renal tubules (RT) with normal hematopoietic tissues (HT) at the interstices of the tubules.

While the kidneys of Ag NPs exposed fish after 24, 48, and 96 h. of exposure (Fig. 6b and e, and 6 h) showed necrotic degeneration (ND); shrinkage in the glomerulus (SH); dilation of Bowman’s space (BS); degeneration in renal corpuscle (DRC). The OP (Fig. 6c, f and i) and BP (Fig. 6d g, and 6j) treated groups shared some alterations with the untreated groups but with less deformation of renal tubules and relatively good maintenance of renal architecture.

### The SEM/EDS spectral and map analysis of OP and BP surfaces

**Fig. 7:**
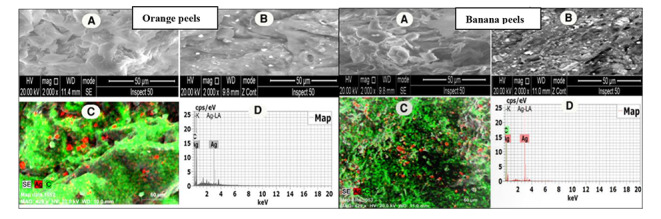
Representative SEM/EDS spectra and map analysis of orange and banana peels. (A: original peels; B: peels after adsorption of Ag-NPs; map analysis of peels after adsorption of Ag-NPs; D: EDS image of peels after adsorption of Ag-NPs)

We are now at the pollutant removal age, so it is no wonder that substantial attempts are being made to establish solutions for reducing the impacts of pollutants on the environment and their biota. Therefore, the aim of the present study was not only to evaluate the toxicological effects of Ag-NPs but also to utilize the adsorptive abilities of OP and BP to reduce the toxicological impacts of Ag-NPs. Based on the SEM/EDS spectra and map analysis, both OP and BP showed a significant ability to adsorb Ag-NPs on their surfaces as shown in Fig. 7.

## Discussion

The nano-scaled size is the influential factor that determines the potency of nano-toxicity (Sharifian et al. 2013). The physical characterizations of the studied Ag-NPs confirmed that all particles were nano-sized with less aggregation potency in water, which may facilitate their access into living organisms through various specialized mechanisms. Such significant features of Ag-NPs may trigger unique toxicological properties as supported by Mao et al. (2018). Silver NPs can enter the fish body, distribute through the bloodstream, and accumulate in different tissues in concentrations exceeding those in their surroundings. While several studies have been performed on Ag-NPs, there is still limited data on their aggregation and impacts on the inner tissues of the exposed fish (Mahmoud et al. 2019). Once Ag-NPs accumulate, tissue-specific adverse effects begin as nano-metals may be distributed equivalently within the tissues but accumulate in significantly different forms (Abdel-Khalek et al. 2020a). The present results showed a great bioaccumulation potency of Ag-NPs in hepatic and renal tissues compared to other tissues in all studied periods. This observation was in agreement with Bacchetta et al. (2017), who observed the same results in juvenile fish, *Piaractus mesopotamicus* after short-term exposure to Ag-NPs. Also, Mahmoud et al. (2019) observed that liver tissues were the major Ag-accumulating tissues in *C. gariepinus.* Due to the lack of the basement membrane in livers and the central location of hepatic circulation, the xenobiotic-hepatocyte interchange was maximized in fish via both intestinal and brachial pathways (Ciji and Bijoy Nandan 2014). The high hepatic metal accumulation may be related to the high rate of metallothioneins (MTs) production, which gradually forms a chelate with metals to prevent potential injury. Furthermore, the liver is a powerful detoxifying organ that is responsible for the mitigation of several metals via sulfur metal protein complex formation (Abdel-Khalek et al. 2020b). Due to the important role of the renal reabsorption and excretion processes, the kidney is considered a potential metal accumulator. Metallic nanoparticles showed an excessive accumulation level in the kidney as their small size accelerates their renal clearance rate (Feng et al. 2018). According to Sivakumar et al. (2012), the excess metals are eliminated rapidly from the bloodstream in the detoxification process (several metals bind with intracellular renal proteins to be converted into a non-toxic soluble form) during the renal excretion phase. The excess mucosal secretions facilitate xenobiotic aggregation in the gills and, consequently, their adsorption and penetration across the surfaces of the gills (Hao et al. 2013). This finding was confirmed in the present work since a significant decrease in Ag content after 48 h. indicated the ability of NPs to penetrate the surface of gills reaching blood circulation. Ag-NPs aggregates (recorded as grey spots during the dissection of the fish) may fix more NPs in the mucus layer and decrease the absorption rate of NPs, as shown by elevated Ag content in the gills after 96 h. The lower Ag content in the skin and muscular tissues compared to the other studied tissues may be due to the absence of metal-binding protein and low metabolic activity of both tissues (Abdel-Khalek et al. 2018). The recorded reduction in RBCs count, Hb content, and Hct level of *Oreochromis niloticus* after exposure to Ag-NPs was in accordance with Thummabancha et al. (2016), who found the same results in *O. niloticus* exposed to Ag-NPs from 1 to 8 weeks. Moreover, Vali et al. (2020) recorded a significant reduction in the RBCs and Hct of common carp (*Cyprinus carpio*) exposed to 25% and 50% of Ag-NPs LC_50_ 96 h. The reduction in RBCs may be due to the direct deleterious effects of Ag nanoparticles which can enter the bloodstream and accumulate in RBCs, or the indirect mechanism for Ag-NPs initiated by the formation of several reactive oxygen species (ROS) that reduce the production of RBCs via the inhibition of DNA synthesis (Ale et al. 2018). The above reasons may cause direct destruction of RBCs, hematopoietic tissue disruption, erythropoiesis disorder, and the development of morphologically altered RBCs. Besides, the damaged erythrocytes can affect both Hb content and Hct levels in exposed fish. Imani et al. (2015) reported that the hypoxic status originated from sharp RBCs reduction may impair aerobic glycolysis and thus reduce the amount of energy necessary for Hb synthesis. Moreover, Kori-Siakpere and Ubogu (2008) stated that the reduced Hct ratio may result from hemodilution and osmoregulatory mechanism disturbance after the histological damage of gills (verified by the histological analysis of the present study). The calculated blood indices showed insignificant changes in most studied groups in all experimental periods. These findings were confirmed by Vali et al. (2020) who found insignificant effects of Ag-NPs on blood indices of *Cyprinus carpio*. Significant MCV elevation and MCHC reduction were observed only in the Ag-NPs exposed group after 48 and 96 h. The enlargement of RBCs correlated with intracellular osmotic disturbance and decreased RBCs count after NPs exposure may be the major causes of these blood index disturbances (Abdel-Khalek et al. 2020a).

Because of their external location, gills are constantly in direct contact with the surrounding environment, which increases their vulnerability to external stressors. Ostaszewska et al. (2016) reported epithelial hypertrophy, hyperplasia, necrosis, lifting, and telangiectasia in *Siberian sturgeon* larvae after exposure to silver and copper nanoparticles. Moreover, many histological alternations in gills such as lamellae fusion, aneurism, and hemorrhage were recorded in *L. rohita* exposed to the waterborne amine-coated silver nanoparticle (Khan et al. 2018). Some of the recorded histological modifications in gills may improve fish resistance against external pollutants. For example, epithelial lifting causes an increase in the distance and time for Ag-NPs to reach the bloodstream. Gill hyperplasia and lamellae fusion can decrease the surface area subjected to Ag-NPs. The decreased surface area of gills may affect the respiratory surface and the oxygen absorption rate. The reduced oxygen consumption will also contribute to blood vessel weakening, blood circulation disruptions, congestion, and aneurysm. The current study found significant deteriorations in liver architecture, including blood cell infiltration, vacuolization, blood congestion, and necrotic degeneration. These changes were in agreement with Khan et al. (2018) who detected congestion and necrosis in hepatic parenchyma with a sharp decrease in hepatic cell size of *L. rohita* treated with the amine-coated silver nanoparticle. The liver of common carp showed deformation in the hepatic blood vessel, necrosis, and accumulation of the color pigmentation after citrate-capped Ag-NPs exposure (Lee et al. 2015). Congestion and RBCs infiltration may be caused by the injurious effect of the accumulated Ag-NPs which weaken the hepatic blood vessels. Ciji and Bijoy Nandan (2014) related hepatic vacuolization to the disproportion between the synthesis and releasing rates of materials produced by the hepatocytes or the excessive cytoplasmic fat deposition. The pyknotic nuclei followed by the degeneration of hepatocytes are a common result of oxidative cell membrane damage (Abdel-Khalek et al. 2020a). The progressive damage of renal tissues recorded in the present study was associated with shrinkage of the glomerulus, dilation of Bowman’s space, degeneration, and deformation of renal corpuscles. These observations agreed with Mahmoud et al. (2019) who observed degeneration in renal tubules, hypertrophy in the glomerulus, necrosis, and shrinkage of the glomerulus in *Clarias gariepinus* exposed to different concentrations and sizes of Ag-NPs. The necrotic degeneration and tubular deformation may be due to the accumulation of inflammatory cells associated with Ag-NPs toxicity. The elevated concentrations of NPs in renal tissues and free radicals produced after NPs toxicity significantly contribute to the injury of the renal tissues. Generally, the severity of the structural damage based on the percentage of the appearance of all recorded alterations (Table 1; supplemental material) was more pronounced in the fish groups that were exposed to Ag-NPs without OP and BP water treatments, especially after 96 h. In addition, the quantitative analysis of the adsorbed Ag-NPs and removal efficiency of both OP and BP were studied in detail in our previous work Abdel-Khalek et al. (2021). According to this study, BP showed higher removal capacities than OP all over the studied intervals. Silver accumulation in all studied tissues was reduced after water treatment consequently an obvious improvement in the studied hematological parameters was recorded. Moreover, the histopathological examination showed that the severity of the recorded alterations (based on the type of alterations, the frequency of appearance, size of affected areas) was more pronounced in the tissues of fish that were exposed to Ag-NPs without any water treatment. This observation is in agreement with Abdel-Khalek et al. (2020a), who showed similar results after using rice husk to reduce the toxicological impacts of the iron and aluminum oxide nanoparticles on *Oreochromis niloticus*. The OP and BP residues can be processed and converted into bio-sorbents due to their chemical composition (high cellulose and other polysaccharide content), large surfaces, wide swelling rate, and excellent mechanical capabilities (Kelly-Vargas et al. 2012). The surface charge of adsorbents is an important parameter in explaining the interaction phenomenon between them and the metal ions. Both fruit peels were reported to have negatively charged surfaces when pH exceeds 7. Therefore, the electrostatic attraction force between the negatively charged surface of the adsorbent and the cationic ions (Ag^+^) led to an increase in silver ion removal efficiency (Pathak et al. 2017). Treatment of water with OP and BP succeeded in decreasing the toxicological impacts of the Ag-NPs, especially in the case of BP water treatment. This may be due to the richness of BP with more surface functional groups, starch, pectin-type compounds, and cellulose compared with OP. Moreover, the existence of additional amine groups in BP also improves its adsorption capacity for metals (Oyewo et al. 2018).

## Conclusion

In summary, the current study found that exposing *O. niloticus* to sub-lethal concentrations of Ag-NPs caused time-dependent accumulative, hematological, and histological effects on various tissues. Both OP and BP showed an efficient adsorbent capacity toward Ag-NPs and offered great potential to improve the health status of the studied fish. Using multiple biomarkers on different fish tissues could pinpoint the overall improvement in fish health following water treatment. The current findings may encourage the application of this water treatment approach on fish farms that depend on waste discharges as a water source.

## Data Availability

The datasets used and/or analyzed during the current study are available from the corresponding author on reasonable request.

## References

[CR1] Abdel-khalek AA, Hamed A, Hasheesh WSF (2021). Does the adsorbent capacity of orange and banana peels toward silver nanoparticles improve the biochemical status of *Oreochromis niloticus*?. Environ Sci Pollut Res.

[CR2] Abdel-Khalek AA, Badran SR, Marie M-AS (2020). The efficient role of rice husk in reducing the toxicity of iron and aluminum oxides nanoparticles in *Oreochromis niloticus*: Hematological, bioaccumulation, and histological endpoints. Water Air Soil Pollut.

[CR3] Abdel-Khalek AA, Zayed HS, Elsayad SM, Zaghloul KH (2020). Assessment of metal pollution impacts on *Tilapia zillii* and *Mugil cephalus* inhabiting Qaroun and Wadi El-Rayan lakes, Egypt, using integrated biomarkers. Environ Sci Pollut Res.

[CR4] Abdel-Khalek AA, Elhaddad E, Mamdouh S, Marie M-AS (2018). The chronic exposure to discharges of Sabal drain induces oxidative stress and histopathological alterations in *Oreochromis niloticus*. Bull Environ Contam Toxicol.

[CR5] Abdel-Khalek AA, Hamed A, Marie MA (2016). The Accumulation Potency of Bulk and Nano Zinc Metal and Their Impacts on the Hematological and Histological Perturbations of *Oreochromis niloticus*. Water Air Soil Pollut.

[CR6] Akpomie KG, Conradie J (2020). Banana peel as a biosorbent for the decontamination of water pollutants. A review. Environ Chem Lett.

[CR8] Ale A, Bacchetta C, Rossi AS, Galdopórpora J, Desimone MF, de la Torre FR, Gervasio S, Cazenave J (2018). Nanosilver toxicity in gills of a neotropical fish: metal accumulation, oxidative stress, histopathology and other physiological effects. Ecotoxicol Environ Safe.

[CR9] Annadurai G, Juang RS, Lee DJ (2003). Adsorption of heavy metals from water using banana and orange peels. Water Sci Technol.

[CR10] APHA (American Public Health Association) (2005) American Water Works Association. Standard methods for the examination of water and wastewater, New York

[CR11] Bacchetta C, Ale A, Simoniello MF, Gervasio S, Davico C, Rossi AS, Desimone MF, Poletta G, Lopez G, Monserrat JM, Cazenave J (2017). Genotoxicity and oxidative stress in fish after a short-term exposure to silver nanoparticles. Ecol Indic.

[CR12] Bernet D, Schmidt H, Meier W, Burkhardt-Holm P, Wahli T (1999). Histopathology in fish: proposal for a protocol to assess aquatic pollution. J Fish Dis.

[CR13] Bhatnagar A, Sillanpää M, Witek-Krowiak A (2015). Agricultural waste peels as versatile biomass for water purification - A review. Chem Eng J.

[CR14] Ciji PP, Bijoy Nandan S (2014). Toxicity of copper and zinc to *Puntius parrah* (Day, 1865). Mar Environ Res.

[CR15] Dacie JV, Lewis SM (1991) Practical hematology. Chuchill. Livigstone, Chap. 5:79

[CR16] Drabkin DL (1964). Spectrophotometric studies: XIV. The crystallographic and optical properties of the hemoglobin of man in comparison with those of other species. J Biol Chem.

[CR17] Feng Q, Liu Y, Huang J, Chen K, Huang J, Xiao K (2018). Uptake, distribution, clearance, and toxicity of iron oxide nanoparticles with different sizes and coatings. Sci Rep.

[CR18] Gupta PK (1977) Haematological Techniques. 4th edition Syndicate, India, pp.231

[CR19] Hao L, Chen L, Hao J, Zhong N (2013). Bioaccumulation and sub-acute toxicity of zinc oxide nanoparticles in juvenile carp (*Cyprinus carpio*): a comparative study with its bulk counterparts. Ecotoxicol Environ Safe.

[CR20] Imani M, Halimi M, Khara H (2015). Effects of silver nanoparticles (Ag-NPs) on hematological parameters of rainbow trout, *Oncorhynchus myk*iss. Comp Clin Pathol.

[CR21] Inshakova E, Inshakov O (2017) World market for nanomaterials: structure and trends. International Conference on Modern Trends in Manufacturing Technologies and Equipment (ICMTMTE). MATEC Web of Conf. 129:1–5. 10.1051/matecconf/201712902013

[CR22] Kelly-Vargas K, Cerro-Lopez M, Reyna-Tellez S, Bandala ER, Sanchez-Salas JL (2012) Biosorption of heavy metals in polluted water, using different waste fruit cortex. Phys Chem Earth parts (A/B/C) 37–39. 10.1016/j.pce.2011.03.006

[CR23] Khan MS, Qureshi NA, Jabeen F, Shakeel M, Asghar MS (2018). Assessment of waterborne amine-coated silver nanoparticle (Ag-NP)-induced toxicity in *labeo rohita* by histological and hematological profiles. Biol Trace Elem Res.

[CR24] Kori-Siakpere O, Ubogu EO (2008). Sublethal haematological effects of zinc on the freshwater fish, Heteroclarias sp. (*Osteichthyes: Clariidae*). Afr J Biotechnol.

[CR25] Lee O, Green JM, Tyler CR (2015). Transgenic fish systems and their application in ecotoxicology. Crit Rev Toxicol.

[CR26] Mahmoud UM, Mekkawy IAA, Naguib M, Sayed AE-DH (2019). Silver nanoparticle–induced nephrotoxicity in *Clarias gariepinus*: physio-histological biomarkers. Fish Physiol Biochem.

[CR27] Mao B-H, Chen Z-Y, Wang Y-J, Yan S-J (2018). Silver nanoparticles have lethal and sublethal adverse effects on development and longevity by inducing ROS-mediated stress responses. Sci Rep.

[CR28] Neugebauer EA, Sans Cartier GL, Wakeford BJ (2000) Methods for the determination of metals in wildlife tissues using various atomic absorption spectrophotometry techniques (Technical Report Series No. 337E). Canadian wildlife service, Headquarters, Hull, Québec, Canada

[CR29] Ostaszewska T, Chojnacki M, Kamaszewski M, Sawosz-Chwalibóg E (2016). Histopathological effects of silver and copper nanoparticles on the epidermis, gills, and liver of *Siberian sturgeon*. Environ Sci Pollut Res.

[CR30] Oyewo OA, Onyango MS, Wolkersdorfer C (2018). Lanthanides removal from mine water using banana peels nanosorbent. Int J Environ Sci Technol.

[CR31] Pathak PD, Mandavgane SA, Kulkarni BD (2017). Fruit peel waste: characterization and its potential uses. Curr Sci.

[CR32] Sarkar B, Jaisai M, Mahanty A, Panda P, Sadique M, Nayak BB, Gallardo G, Thakur D, Bhattacharjee S, Dutta J (2015). Optimization of the sublethal dose of silver nanoparticle through evaluating its effect on intestinal physiology of Nile tilapia (*Oreochromis niloticus L.*). J Environ Sci Heal-Part A Toxic/Hazard Sub Environ Eng.

[CR33] Sharifian M, Khani F, Khosravi K, Khalili M, Hedayati A (2013). Sublethal effect of nanosilver on the structure of gill of Caspian roach (*Rutilus rutilus caspicus*) fingerlings. Int J Aquat Biol.

[CR34] Singh NB, Nagpal G, Agrawal S, Rachna (2018). Water purification by using Adsorbents: A Review. Environ Technol Innovat.

[CR35] Sivakumar S, Khatiwada CP, Sivasubramanian J (2012). Bioaccumulations of aluminum and the effects of chelating agents on different organs of *Cirrhinus mrigala*. Environ Toxicol Pharmacol.

[CR36] Thummabancha K, Onparn N, Srisapoome P (2016). Analysis of hematologic alterations, immune responses and metallothionein gene expression in Nile tilapia (*Oreochromis niloticus*) exposed to silver nanoparticles. J Immunotoxicol.

[CR37] Tunçsoy M, Duran S, Ay Ö, Cicik B, Erdem C (2017). Effects of copper oxide nanoparticles on antioxidant enzyme activities and on tissue accumulation of *Oreochromis niloticus*. Bull Environ Contam Toxicol.

[CR38] Vali S, Mohammadi G, Tavabe KR, Moghadas F, Naserabad SS (2020). The effects of silver nanoparticles (Ag-NPs) sublethal concentrations on common carp (*Cyprinus carpio)*: Bioaccumulation, hematology, serum biochemistry and immunology, antioxidant enzymes, and skin mucosal responses. Ecotoxicol Environ Safe.

